# Histological Characteristics of Intracranial Atherosclerosis in a Chinese Population: A Postmortem Study

**DOI:** 10.3389/fneur.2017.00488

**Published:** 2017-09-25

**Authors:** Wen Jie Yang, Mark Fisher, Lu Zheng, Chun Bo Niu, Annlia Paganini-Hill, Hai Lu Zhao, Yun Xu, Ka Sing Wong, Ho Keung Ng, Xiang Yan Chen

**Affiliations:** ^1^Department of Medicine and Therapeutics, Chinese University of Hong Kong, Shatin, Hong Kong; ^2^Department of Neurology, University of California Irvine, Irvine, CA, United States; ^3^Department of Pathology & Laboratory Medicine, University of California Irvine, Irvine, CA, United States; ^4^Department of Pathology, China-Japan Union Hospital Affiliated to Jilin University, Jilin, China; ^5^Center for Diabetic Systems Medicine, Guangxi Key Laboratory of Excellence, Guilin Medical University, Guilin, China; ^6^Department of Neurology, Affiliated Drum Tower Hospital of Nanjing University Medical School, Nanjing, China; ^7^Department of Anatomical and Cellular Pathology, Chinese University of Hong Kong, Shatin, Hong Kong

**Keywords:** intracranial atherosclerosis, pathology, anterior circulation, posterior circulation, intraplaque hemorrhage, thrombus

## Abstract

**Background:**

Anterior and posterior circulation atherosclerosis differ in vascular risk factors and stroke mechanisms. However, few studies have compared the pathological features between these lesions. Using a series of intracranial artery specimens, we characterized the intracranial atherosclerotic lesions and compared pathological features among different arteries of the intracranial vasculature.

**Methods:**

Intracranial large arteries of 32 consecutively recruited autopsy cases of Chinese adults aged 45 years or older were examined pathologically using routine histology and immunostaining, to characterize the pathological features of the atherosclerotic lesions. We analyzed middle cerebral arteries (MCAs) (both left and right), vertebral arteries (VAs) (side more affected), and basilar arteries (BAs).

**Results:**

Progressive atherosclerotic lesions were present in 91(71%) of the 128 arteries examined. Features of complicated plaques were infrequently detected: plaque hemorrhage was encountered in 12%, neovasculature in 12%, lumen thrombi in 13%, macrophage infiltration in 20%, and calcification in 25% of arteries. Luminal narrowing of MCA was the most severe, followed by VA; the BA least stenotic (37 ± 25 vs. 30 ± 24 vs. 20 ± 20%, all *p* < 0.05). MCA had more eccentric (vs. concentric) plaques than VA (69 vs. 25%, *p* = 0.003) and BA (69 vs. 38%; *p* = 0.03). Lumen thrombi were more frequent in BA, and calcification most commonly occurred in VA atherosclerotic lesions.

**Conclusion:**

Intracranial atherosclerotic plaques were commonly present in this sample, but the lesions generally lacked features of complicated plaques. MCA lesions had demonstrable differences compared with VA and BA lesions. Further studies are needed to determine whether these characteristics indicate a distinctive atherosclerotic phenotype for the intracranial vasculature.

## Introduction

Intracranial atherosclerotic disease (ICAD) is gaining greater clinical prominence as one of the most common causes of ischemic stroke worldwide, especially in Asians (1, 2). Current understanding of ICAD has been advanced by several imaging modalities (3), especially high-resolution magnetic resolution imaging (HRMRI) used to visualize intracranial vessel wall pathology (4–6). Although recent advances in HRMRI have made it possible to provide information on abnormalities within the intracranial vessel, use of HRMRI to characterize ICAD with respect to plaque components and vulnerability is limited due to lack of histologic validation (7–11). Better understanding of pathological characteristics of ICAD may have important consequences for interpretation of plaque vessel wall imaging and provide insights into the pathogenesis of ischemic stroke.

Recent studies demonstrated that anatomical location is an important determinant of arterial morphometric characteristics (12, 13), and anterior circulation atherosclerosis differs from posterior circulation in prevalence of risk factors and stroke mechanisms (14–17). The purpose of the present study was to investigate histologic features of intracranial atherosclerosis in a series of postmortem specimens taken from Chinese subjects and to identify differences between lesions in the anterior vs. posterior circulation. We hypothesized that substantial differences are identifiable between plaques in the anterior vs. posterior circulation.

## Patients and Methods

### Participants

All large intracranial arteries were retrieved from 32 autopsied specimens from December 2003 to June 2005 at the Prince of Wales Hospital, Hong Kong (18), who were indicated for clinical autopsy to investigate the uncertain cause of death. Consent forms were signed by the patients’ relatives for performing routine clinical autopsy. Our protocol was approved by the Joint Chinese University of Hong Kong-New Territories East Cluster Clinical Research Ethics Committee (The Joint CUHK-NTEC CREC) for retrospectively collecting the artery specimens from the autopsy cases. The autopsy examination was performed by certified pathologists who were blinded to the aims of this study. Data of clinical characteristics and cause of death were collected from hospital electronic patient records. The causes of death were as follows: CVD (i.e., coronary artery disease, hypertensive heart disease, ischemic stroke, and brain hemorrhage), *n* = 13 (41%); infection or sepsis, *n* = 3 (9%); other natural causes (i.e., hepatitis), *n* = 13 (41%); unnatural causes (i.e., suicides, accidents), *n* = 3 (9%).

### Histological Staining

Intracranial large arteries [bilateral middle cerebral artery (MCA), bilateral vertebral artery (VA), and basilar artery (BA)] were extracted for each of the 32 subjects. Each artery was divided into three equal parts. Each part was then dissected and decalcified overnight in 10% formic acid, followed by perfusion fixation in fresh 30% formaldehyde. Serial sections of the isolated arteries were cut transversely at 4-mm intervals, embedded in paraffin. Bilateral VA, paralleling left and right VA for individual cases, were embedded into one paraffin block together, without special labeling left or right ride. For an individual autopsy case, the side of VA with a more severe degree of atherosclerotic lesions was used in the analysis. Sections 5-µm thick were cut and stained with hematoxylin–eosin (H&E) and Victoria blue. Immunohistochemical staining was performed to evaluate the degree of macrophage infiltration, using standard avidin-biotin techniques and commercially available antibodies for CD68 (Abcam, Cambridge, MA, USA) at a dilution of 1: 200.

### Plaque Analysis

Two pathologists (Chun Bo Niu and Hai Lu Zhao), who were blinded to clinical data, examined the histological sections from all segments using standard light microscopy. For each large artery, only the atherosclerotic lesion with the maximum grade of phenotype was subjected to histological and immunohistochemical evaluations. For the 32 autopsy cases, the most stenotic locations from each of the left MCA, right MCA, VA (choosing the artery with more severe lesions), and BA were analyzed for a total of 128 intracranial arteries.

According to the revised American Heart Association criteria (19), the lesions were classified into three groups: (1) disease-free, with normal intima; (2) pre-atherosclerotic intimal lesions showing accumulation of smooth muscle cells (intimal thickening), or luminal accumulation of foam cells (intimal xanthoma); these lesions commonly occur soon after birth and may regress in the later life; (3) progressive atherosclerotic lesions showing extracellular lipid accumulation without necrosis (pathologic intimal thickening), well-formed necrotic core with an overlying fibrous cap (fibrous cap atheroma and thin fibrous cap atheroma), and large areas of calcification (fibrocalcific plaque).

Lesions in which the atherosclerotic plaques were distributed along the entire circumference of the internal elastic laminar were identified as concentric plaques, and those with a disease-free wall present were defined as eccentric plaques, according to prior work (20). The occurrence of plaque complications, including intraplaque hemorrhage (presence of erythrocytes and/or hemosiderin), neovasculature, thrombus, macrophage infiltration, and calcification, were then analyzed and recorded as absent or present.

The histological sections were photographed with a Leica DC 200 digital microscope. The degree of luminal narrowing was measured using Image-Pro Plus according to the method described by Leung et al. (21). The internal elastic membrane in Victoria blue staining was used as the original boundary of the lumen. The internal elastic lamina was traced, and the perimeter of the area bounded by the internal elastic lamina was recorded as “*P*.” The original luminal area “*A*” was determined by the formula: *A* = *P*^2^/4π. The area of atherosclerotic plaque was then traced and determined by the software automatically (recorded as “Ai”), and the percentage of luminal narrowing was determined by the formula (Ai/*A*)*100%. This method eliminated errors due to luminal collapse that might occur during processing.

### Statistical Analysis

Data analyses were conducted with the SPSS 20.0 software package (SPSS, Inc., USA). Comparisons among MCA, VA, and BA groups were assessed by McNemar’s test for categorical data and paired-samples *t*-test for continuous data. A value of *p* < 0.05 was considered to be statistically significant.

## Results

### General Features of Intracranial Atherosclerosis

The 32 cases had a median age of 71 years (range 45–97 years) and most were men (*n* = 23, 72%). Nine cases (28%) were smokers. Hypertension was present in nine cases (28%) and diabetes in six (19%). Cardiovascular diseases including ischemic heart disease, ischemic stroke, and hemorrhagic stroke were found in 9 (28%), 14 (44%), and 2 (6%) cases, respectively (Table 1). According to brain MRI and/or autopsy findings, 11 cases showed brain infarct in MCA territories while only 1 case (with atrial fibrillation) had an infarct in posterior circulation territories.

**Table 1:** Clinical characteristics of 32 autopsy cases.

	Total*
Age (years)	71 [45–97]
Male	23 (72%)
Smoker	9 (28%)
Hypertension	9 (28%)
Diabetes	6 (19%)
Ischemic heart disease	9 (28%)
Ischemic stroke	14 (44%)
Hemorrhagic stroke	2 (6%)

*Median values with the interquartile range, median [IQR] or number of patients with the percentage, *n* (%) is shown.

**Figure 1:**
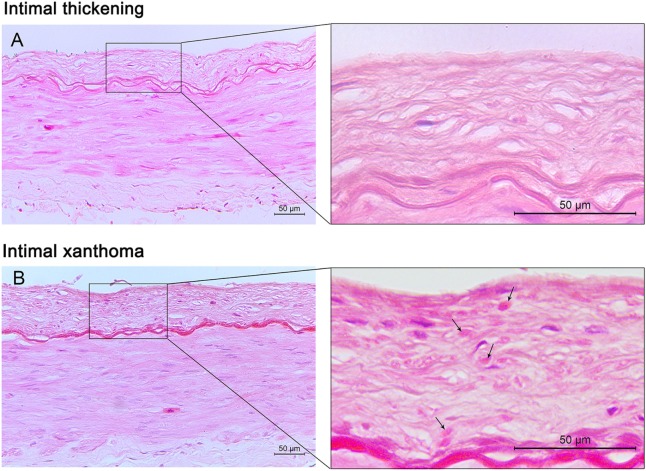
Pre-atherosclerotic intimal lesions. Intimal thickening **(A)** consists mainly of smooth muscle cells in a proteoglycan-rich matrix. Intimal xanthoma **(B)** displays intimal thickening with isolated foam cells (arrows). Hematoxylin and eosin (H&E) staining; original magnification: 10× for original images, 40× for inserted images.

**Figure 2:**
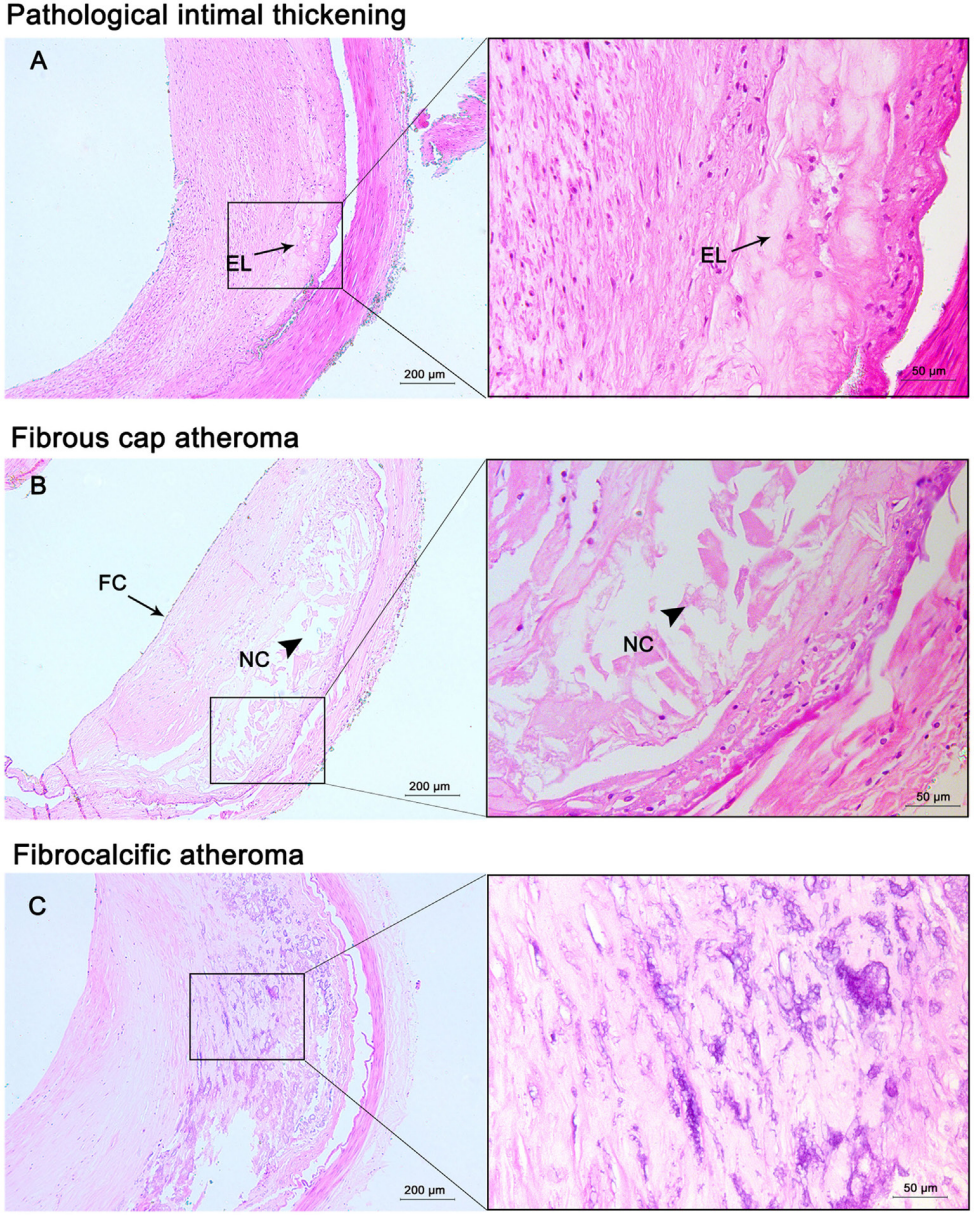
Progressive atherosclerotic lesions. Pathological intima thickening **(A)** has some extracellular lipid (EL) present deep in the lesion without true necrosis. Fibrous cap atheroma **(B)** has a well-formed necrotic core (NC) containing lipids with an overlying thick fibrous cap (FC). Fibrocalcific plaques **(C)** are heavily calcified lesions with or without a necrotic core. Hematoxylin and eosin (H&E) staining; original magnification: 5× for original images, 20× for inserted images.

**Figure 3:**
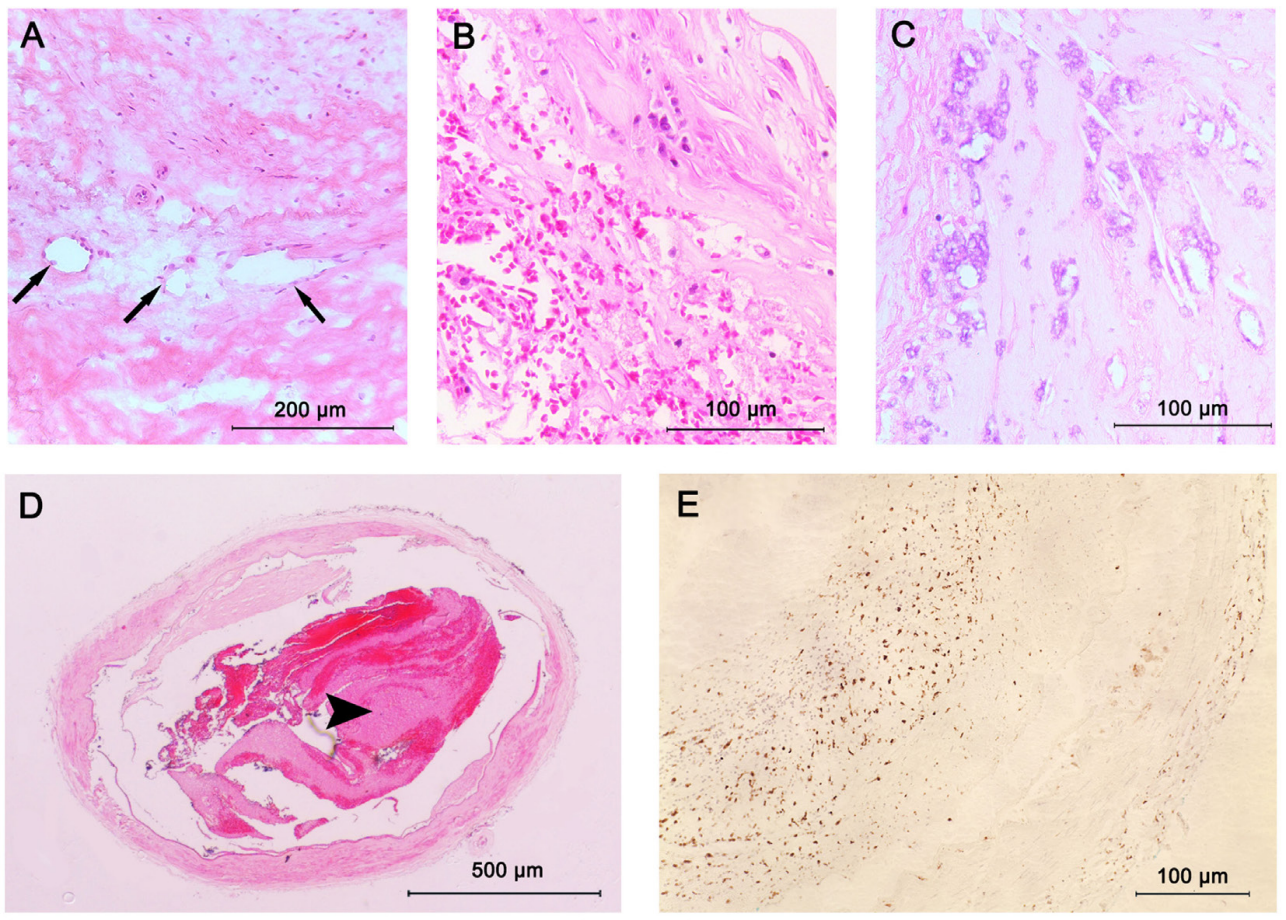
Plaque components in intracranial atherosclerosis. **(A)** Intraplaque neovasculature (arrows); **(B)** intraplaque hemorrhage; **(C)** large areas of calcification seen as purple morula; **(D)** lumen thrombus (arrowhead); stained with hematoxylin and eosin (H&E); **(E)** macrophage infiltration; stained with CD68 antibodies.

A total of 128 large arteries from 32 autopsy cases were analyzed, including both left and right MCA, the more affected VA, and BA for each patient. Among all the arteries, 37 (29%) had pre-atherosclerotic intimal lesions, whereas 91 (71%) showed progressive atherosclerotic lesions, including 15 (12%) pathologic intimal thickening, 72 (56%) fibrous cap atheroma, and 4 (3%) fibrocalcific plaque (Figures 1 and 2). Significant atheromatous narrowing of intracranial vessels was infrequently seen, with only 23 segments (18%) affected by severe atherosclerotic lesions (>50% luminal narrowing), while 68 arteries (53%) exhibited moderate stenosis (10–50% luminal narrowing), with a mean ± SD stenosis area of 29 ± 23%. Components of complicated plaques were relatively uncommon, with plaque hemorrhage encountered in 15 (12%), neovasculature in 15 (12%), lumen thrombus in 17 (13%), macrophage infiltration in 26 (20%), and calcification in 32 (25%) of 128 arteries (Figure 3). Ulceration and calcified nodule were not detected in any artery.

### Differences among MCA, VA, and BA Atherosclerosis

Plaque morphology and components were compared among MCA, VA and BA. Statistical analysis comparing left and right MCA showed no significant differences. We therefore chose the MCA with more severe lesions for each patient (32 MCAs) and compared the plaque characteristics with 32 VAs and 32 BAs. As shown in (Table 2), the mean area of luminal stenosis rose from 20% in the BA group to 30% in VA group and 37% in MCA group (all *p* < 0.05). Compared with posterior circulation arteries (VA and BA), anterior circulation arteries (MCA) had a higher prevalence of eccentric lesions (69 vs. 25% and 38%; *p* = 0.003 and *p* = 0.03, respectively) and a lower prevalcne of lumen thrombi (6 vs. 16%, NS and 28%, *p* = 0.04). VA plaques showed a higher frequency of calcification compared with BA plaques (41 vs. 16%, *p* = 0.04). Plaque phenotype did not show significant difference between anterior and posterior circulation arteries. Other components were detected similarly between anterior and posterior circulation plaques.

**Table 2:** Comparisons of plaque characteristics among middle cerebral artery (MCA), vertebral artery (VA), and basilar artery (BA) groups.

	MCA (*n* = 32)	VA (*n* = 32)	BA (*n* = 32)	*p*-Value^#^	*p*-Value^##^	*p*-Value^###^
Plaque phenotype				0.109	0.07	1.00
Pre-atherosclerotic intimal lesions	6 (19%)	12 (38%)	12 (38%)			
Progressive atherosclerotic lesions	26 (81%)	20 (62%)	20 (62%)			
Area stenosis	37 ± 25%	30 ± 24%	20 ± 20%	0.048*	<0.001*	0.001*
Distribution				0.003*	0.03*	0.42
Eccentric	22 (69%)	8 (25%)	12 (38%)			
Concentric	10 (31%)	24 (75%)	20 (62%)			
Intraplaque hemorrhage	3 (9%)	5 (16%)	3 (9%)	0.69	1.00	0.63
Neovasculature	6 (19%)	3 (9%)	3 (9%)	0.51	0.45	1.00
Lumen thrombi	2 (6%)	5 (16%)	9 (28%)	0.45	0.04*	0.29
Macrophages	8 (25%)	7 (22%)	7 (22%)	1.00	1.00	1.00
Calcification	9 (28%)	13 (41%)	5 (16%)	0.61	0.23	0.04*

*^#^MCA vs. VA; ^##^MCA vs. BA; ^###^VA vs. BA*.

*p< 0.05

## Discussion

Based on this postmortem study, the intracranial arteries commonly exhibit atherosclerotic lesions in Chinese adults, but the degree of luminal narrowing is low and features of complicated plaques are infrequent. We found that progressive atherosclerotic plaques and eccentric lesions occur more commonly in anterior circulation. The extent of area stenosis varies among large cerebral arteries, with MCA the most severe, followed by VA, and BA the least. Lumen thrombi are more commonly observed in posterior circulation, and calcification is most prevalent in VA plaques.

Previous studies have shown that plaque formation occurs throughout the arteries of Circle of Willies and involves arteries contralateral to brain infarct, although the degree of stenosis, neovasculature, and lipid core were higher in ipsilateral arteries (18, 22–27). In the present study, we found that progressive intracranial atherosclerosis was very common in Chinese adults involving 71% of large cerebral arteries. But the prevalence of intracranial plaques was lower in Caucasian population, with 40% of ICAS reported in Caucasian patients aged 60 or older (25) and 15% of advanced plaques in another autopsy studies of 196 Caucasian cases (26).

Although atherosclerotic lesions in intracranial arteries were prevalent, the features of complicated plaque were uncommon (28). A prior autopsy study investigating BA atherosclerosis in patients with a history of stroke depicted a relatively benign histopathological profile, with rare neovasculature, infrequent inflammation, and rare ulceration and rupture (29). Consistent with those findings, we found that plaque complications such as intraplaque hemorrhage, neovasculature, and infiltration of macrophages were less commonly detected when compared with other vascular beds. Also note that plaque ulceration and calcified nodules were not observed in our specimens. Intraplaque hemorrhage was present in only 12% of intracranial arteries, less than in carotid arteries (19–97% in symptomatic patients and 7–91% in asymptomatic patients) (30, 31). However, this prevalence in carotid artery studies was primarily in carotid endarterectomy patients, a highly select population with the most severe degree of atherosclerosis. The few studies evaluating intracranial intraplaque hemorrhage using HRMRI have shown substantial variation in its prevalence. Intraplaque hemorrhage was identified in 10% of high-grade (>70%) stenotic MCA (32), 17% of > 50% stenotic MCA (33), and 42% of BA (34). Nonetheless, the intracranial intraplaque hemorrhage predicted by MRI has not been validated by standard histology. Note, however, that our autopsy cases were from the general population and few were stroke patients; subjects recruited in HRMRI studies were stroke patients affected by intracranial atherosclerosis. Calcification was prevalent in VA, consistent with the computed tomography findings showing that intracranial VA is the second most common artery affected by calcification after internal carotid artery (35, 36).

A notable finding was that luminal stenosis was significantly different among large intracranial arteries, with MCA the most severe, followed by VA, and BA was the least stenotic. This is consistent with previous reports that MCA was the most commonly affected site by intracranial atherosclerotic lesions in Asians (1, 37, 38). Additionally, we found that the majority of MCA plaques were eccentric lesions, while more VA and BA plaques were concentric. This was consistent with a recent autopsy study in a racially mixed population (39).

This study has some limitations. First, the sample size was relatively small, considering the complexity of atherosclerosis phenotypes and the variations of plaque features between anterior and posterior circulation. Second, we did not include analysis of internal carotid arteries. Finally, plaque features were largely identified based on hematoxylin–eosin staining, which may limit the detection of plaque components.

Intracranial atherosclerosis lesions are commonly present in Chinese adults, with MCA tending to have more severe luminal stenosis and higher prevalence of eccentric lesions. Pathological data of the cerebral arteries may provide reference values for better interpretation of signal changes in HRMRI. Variations of plaque features between anterior and posterior cerebral circulation suggest potentially distinct treatment strategies for stroke patients with brain infarctions occurring in anterior and posterior circulation. Further studies are needed to confirm whether the observed low frequency of complicated plaques represents a distinct phenotype of intracranial atherosclerosis.

## Ethics Statement

This study was carried out in accordance with the recommendations of “The Joint Chinese University of Hong Kong-New Territories East Cluster Clinical Research Ethics Committee (The Joint CUHK-NTEC CREC).” The protocol was approved by the “The Joint CUHK-NTEC CREC.” Consent forms were signed by the subjects’ relatives.

## Author Contributions

WY, LZ, and AP-H were involved in data collection, analysis interpretation, and/or manuscript writing. MF, YX, KW, and XC were responsible for the conception of the study, study design, manuscript writing, and/or final approval. CN and HZ were responsible for the pathological slides review. HN was responsible for the autopsy examination. All authors approved the final report.

## Conflict of Interest Statement

The authors declare that the research was conducted in the absence of any commercial or financial relationships that could be construed as a potential conflict of interest.
